# Supplementary material for: MetaRibo-Seq measures translation in microbiomes
Source: Nat Commun. 2020 Jun 29;11:3268. doi: 10.1038/s41467-020-17081-z (PMC7324362; doi:10.1038/s41467-020-17081-z)
Supplement: Supplementary file 10 — Supplementary Data 7 [file 41467_2020_17081_MOESM10_ESM.zip › File2/Confidence_High_Taxonomy/259702_out.krona.html]

Javascript must be enabled to view this page.

members
magnitude
magnitudeUnassigned
count
unassigned
taxon
rank

259702\_out

388

388
superkingdom
2

388
phylum
976

200643
388
class

order
388
171549

387
family
815

816
genus
387
37

SRS011084\_contig\_number\_21409SRS013687\_contig\_number\_27417SRS013800\_contig\_number\_12513SRS013951\_contig\_number\_27556SRS015794\_contig\_number\_883SRS015960\_contig\_number\_21965SRS017307\_contig\_number\_15799SRS017375\_contig\_number\_851SRS019445\_contig\_number\_25845SRS020394\_contig\_number\_1777SRS021153\_contig\_number\_936SRS022071\_contig\_number\_30357SRS022524\_contig\_number\_10829SRS022609\_contig\_number\_40573SRS024132\_contig\_number\_41521SRS024331\_contig\_number\_11608SRS024435\_contig\_number\_42702SRS045713\_contig\_number\_10370SRS047433\_contig\_number\_11330SRS049773\_contig\_number\_35411SRS050941\_contig\_number\_15037SRS051031\_contig\_number\_4973SRS053356\_contig\_number\_contig-100\_459.267974SRS054352\_contig\_number\_16143SRS056259\_contig\_number\_6866SRS056273\_contig\_number\_2921SRS056519\_contig\_number\_960SRS058723\_contig\_number\_1581SRS063985\_contig\_number\_4938SRS077127\_contig\_number\_15326SRS1041095\_contig\_number\_1608SRS1054691\_contig\_number\_17006SRS1055022\_contig\_number\_19538SRS140513\_contig\_number\_9325SRS148319\_contig\_number\_13838SRS149181\_contig\_number\_6276SRS893382\_contig\_number\_673


SRS011239\_contig\_number\_9090
310298
species
1


SRS017103\_contig\_number\_35044SRS056537\_contig\_number\_contig-100\_152.383067SRS077392\_contig\_number\_35447SRS143523\_contig\_number\_20883
species
4
671267


SRS147425\_contig\_number\_2184
556259
1
species

4
species
310297

SRS019381\_contig\_number\_1561SRS023583\_contig\_number\_10596SRS065176\_contig\_number\_2793SRS145308\_contig\_number\_633


SRS020233\_contig\_number\_45713
2292284
1
species


SRS014979\_contig\_number\_30174SRS024075\_contig\_number\_1577SRS075963\_contig\_number\_contig-100\_298.298SRS076876\_contig\_number\_445SRS104084\_contig\_number\_10629SRS893300\_contig\_number\_11974SRS893382\_contig\_number\_5446
820
species
7

1
species
1262750

SRS055966\_contig\_number\_218


SRS011302\_contig\_number\_23866SRS011405\_contig\_number\_11767SRS012902\_contig\_number\_78SRS012969\_contig\_number\_11582SRS013158\_contig\_number\_763SRS013382\_contig\_number\_535SRS013521\_contig\_number\_3892SRS013638\_contig\_number\_5014SRS013965\_contig\_number\_28005SRS014235\_contig\_number\_34639SRS014287\_contig\_number\_17424SRS014313\_contig\_number\_4129SRS014412\_contig\_number\_17521SRS014459\_contig\_number\_2405SRS014534\_contig\_number\_614SRS014736\_contig\_number\_971SRS014855\_contig\_number\_11973SRS014948\_contig\_number\_17831SRS015065\_contig\_number\_929SRS015190\_contig\_number\_3497SRS015217\_contig\_number\_28787SRS015369\_contig\_number\_1897SRS015431\_contig\_number\_29500SRS015486\_contig\_number\_321SRS015578\_contig\_number\_1753SRS015579\_contig\_number\_5081SRS015663\_contig\_number\_14485SRS015694\_contig\_number\_19411SRS015782\_contig\_number\_949SRS015854\_contig\_number\_10562SRS016056\_contig\_number\_2451SRS016095\_contig\_number\_11916SRS016203\_contig\_number\_8547SRS016335\_contig\_number\_29007SRS016495\_contig\_number\_10608SRS016629\_contig\_number\_1005SRS016753\_contig\_number\_1399SRS016840\_contig\_number\_1464SRS016897\_contig\_number\_44SRS016954\_contig\_number\_3423SRS017191\_contig\_number\_12401SRS017433\_contig\_number\_724SRS017521\_contig\_number\_29069SRS017745\_contig\_number\_467SRS017821\_contig\_number\_21303SRS017859\_contig\_number\_1312SRS018351\_contig\_number\_5202SRS018623\_contig\_number\_452SRS018817\_contig\_number\_13381SRS018888\_contig\_number\_8314SRS018936\_contig\_number\_8368SRS019068\_contig\_number\_56509SRS019178\_contig\_number\_1168SRS019267\_contig\_number\_3056SRS019397\_contig\_number\_4037SRS019496\_contig\_number\_9637SRS019601\_contig\_number\_2155SRS019693\_contig\_number\_7678SRS019808\_contig\_number\_20524SRS019968\_contig\_number\_9178SRS020328\_contig\_number\_1400SRS020508\_contig\_number\_71SRS020869\_contig\_number\_30345SRS021484\_contig\_number\_895SRS022713\_contig\_number\_627SRS023829\_contig\_number\_121SRS023971\_contig\_number\_629SRS024009\_contig\_number\_1637SRS024625\_contig\_number\_544SRS043411\_contig\_number\_11196SRS043701\_contig\_number\_1061SRS044535\_contig\_number\_13001SRS045004\_contig\_number\_532SRS045244\_contig\_number\_726SRS045645\_contig\_number\_1254SRS045826\_contig\_number\_19111SRS046068\_contig\_number\_1391SRS046369\_contig\_number\_9544SRS046502\_contig\_number\_10042SRS046717\_contig\_number\_390SRS046810\_contig\_number\_4SRS047014\_contig\_number\_28117SRS047044\_contig\_number\_1261SRS048164\_contig\_number\_12488SRS049402\_contig\_number\_2503SRS049712\_contig\_number\_16616SRS049900\_contig\_number\_4744SRS049995\_contig\_number\_33154SRS050026\_contig\_number\_1704SRS050422\_contig\_number\_26621SRS050520\_contig\_number\_67SRS050752\_contig\_number\_11069SRS050974\_contig\_number\_1804SRS050998\_contig\_number\_11452SRS051610\_contig\_number\_10901SRS052576\_contig\_number\_112SRS052697\_contig\_number\_554SRS053087\_contig\_number\_715SRS053214\_contig\_number\_470SRS053335\_contig\_number\_9383SRS053649\_contig\_number\_14126SRS054059\_contig\_number\_5136SRS055017\_contig\_number\_29921SRS057480\_contig\_number\_8973SRS058268\_contig\_number\_3SRS062427\_contig\_number\_1133SRS062654\_contig\_number\_4142SRS063127\_contig\_number\_1252SRS063190\_contig\_number\_2721SRS063370\_contig\_number\_5690SRS063489\_contig\_number\_5625SRS063518\_contig\_number\_15631SRS063813\_contig\_number\_3797SRS063928\_contig\_number\_652SRS064232\_contig\_number\_2778SRS064557\_contig\_number\_5089SRS064757\_contig\_number\_17650SRS064973\_contig\_number\_80SRS065397\_contig\_number\_1205SRS075398\_contig\_number\_1429SRS075716\_contig\_number\_991SRS075821\_contig\_number\_10910SRS075984\_contig\_number\_15657SRS076929\_contig\_number\_21940SRS076976\_contig\_number\_3840SRS077294\_contig\_number\_4131SRS077454\_contig\_number\_10494SRS077502\_contig\_number\_2149SRS077641\_contig\_number\_18962SRS077730\_contig\_number\_2246SRS077849\_contig\_number\_12097SRS077991\_contig\_number\_60SRS078176\_contig\_number\_18435SRS078665\_contig\_number\_2408SRS097920\_contig\_number\_16878SRS097958\_contig\_number\_17875SRS098061\_contig\_number\_4252SRS098571\_contig\_number\_28088SRS098827\_contig\_number\_1112SRS098881\_contig\_number\_4057SRS100907\_contig\_number\_8651SRS101376\_contig\_number\_1946SRS101433\_contig\_number\_7522SRS103987\_contig\_number\_4388SRS1041031\_contig\_number\_25141SRS1041032\_contig\_number\_16761SRS1041036\_contig\_number\_1920SRS1041037\_contig\_number\_13038SRS1041039\_contig\_number\_19984SRS1041090\_contig\_number\_3566SRS1041091\_contig\_number\_16666SRS1041092\_contig\_number\_8877SRS1041116\_contig\_number\_7538SRS1041118\_contig\_number\_1959SRS1041129\_contig\_number\_12137SRS1041130\_contig\_number\_1095SRS1041134\_contig\_number\_11367SRS1041136\_contig\_number\_18192SRS1041137\_contig\_number\_29252SRS1041138\_contig\_number\_4058SRS1041139\_contig\_number\_1502SRS1041140\_contig\_number\_8447SRS1041141\_contig\_number\_11820SRS1041142\_contig\_number\_1721SRS1041143\_contig\_number\_8400SRS1041144\_contig\_number\_1533SRS1041147\_contig\_number\_863SRS1041157\_contig\_number\_4339SRS1041164\_contig\_number\_1488SRS104327\_contig\_number\_3266SRS104400\_contig\_number\_12274SRS104693\_contig\_number\_734SRS104912\_contig\_number\_4468SRS104975\_contig\_number\_9910SRS1054928\_contig\_number\_1747SRS1055038\_contig\_number\_2820SRS1055049\_contig\_number\_10331SRS1055056\_contig\_number\_10796SRS1055069\_contig\_number\_620SRS1055076\_contig\_number\_9490SRS1055083\_contig\_number\_467SRS1055095\_contig\_number\_2308SRS142503\_contig\_number\_28090SRS142542\_contig\_number\_6717SRS142599\_contig\_number\_5638SRS142890\_contig\_number\_6813SRS142923\_contig\_number\_2402SRS142980\_contig\_number\_5344SRS143070\_contig\_number\_3555SRS143085\_contig\_number\_1664SRS143148\_contig\_number\_21834SRS143181\_contig\_number\_12603SRS143342\_contig\_number\_17705SRS143372\_contig\_number\_1693SRS143722\_contig\_number\_4258SRS143876\_contig\_number\_12613SRS143895\_contig\_number\_33814SRS143991\_contig\_number\_5839SRS144135\_contig\_number\_33730SRS144183\_contig\_number\_35242SRS144297\_contig\_number\_7444SRS144506\_contig\_number\_8811SRS144603\_contig\_number\_3726SRS144714\_contig\_number\_22055SRS145497\_contig\_number\_2815SRS146813\_contig\_number\_11257SRS147039\_contig\_number\_3923SRS147271\_contig\_number\_8384SRS147346\_contig\_number\_11598SRS147377\_contig\_number\_3372SRS147557\_contig\_number\_30128SRS147614\_contig\_number\_4844SRS147653\_contig\_number\_1937SRS147766\_contig\_number\_1074SRS148091\_contig\_number\_9794SRS148159\_contig\_number\_7918SRS148196\_contig\_number\_17465SRS148253\_contig\_number\_3681SRS148424\_contig\_number\_5853SRS148511\_contig\_number\_18446SRS148721\_contig\_number\_32139SRS148784\_contig\_number\_22388SRS148817\_contig\_number\_9453SRS149075\_contig\_number\_1237SRS149244\_contig\_number\_2148SRS150029\_contig\_number\_2294SRS892882\_contig\_number\_12767SRS893170\_contig\_number\_1409SRS893172\_contig\_number\_10428SRS893187\_contig\_number\_11365SRS893252\_contig\_number\_16996SRS893253\_contig\_number\_265SRS893256\_contig\_number\_302SRS893259\_contig\_number\_1520SRS893270\_contig\_number\_14955SRS893279\_contig\_number\_5944SRS893285\_contig\_number\_1092SRS893292\_contig\_number\_1531SRS893295\_contig\_number\_10817SRS893327\_contig\_number\_3312SRS893341\_contig\_number\_2750SRS893342\_contig\_number\_8142SRS893358\_contig\_number\_3050SRS893366\_contig\_number\_17629SRS893373\_contig\_number\_9314SRS893378\_contig\_number\_497SRS893380\_contig\_number\_349SRS893383\_contig\_number\_18643SRS971276\_contig\_number\_32453
species
249
821

species
1
2292923

SRS016381\_contig\_number\_2330


SRS017247\_contig\_number\_1654SRS021219\_contig\_number\_1554SRS023914\_contig\_number\_787SRS051882\_contig\_number\_644SRS054905\_contig\_number\_4998SRS058070\_contig\_number\_11445SRS148970\_contig\_number\_123
species
7
818


SRS045195\_contig\_number\_6769SRS148874\_contig\_number\_7704
457395
2
species

2292914
species
2

SRS017701\_contig\_number\_15334SRS078419\_contig\_number\_20773


SRS018575\_contig\_number\_145
1
species
1739517

1
species
1263042

SRS104636\_contig\_number\_1414


SRS100021\_contig\_number\_7846SRS105082\_contig\_number\_345
338188
species
2


SRS098717\_contig\_number\_5772
376806
1
species

817
5
species

SRS050299\_contig\_number\_9447SRS075878\_contig\_number\_3077SRS098644\_contig\_number\_7484SRS140492\_contig\_number\_618SRS893288\_contig\_number\_111

46506
5
species

SRS018836\_contig\_number\_20835SRS042966\_contig\_number\_12976SRS043001\_contig\_number\_9094SRS057478\_contig\_number\_373SRS1041133\_contig\_number\_19950


SRS024492\_contig\_number\_5951
162156
species
1

1
species
457391

SRS018541\_contig\_number\_9336

species
6
47678

SRS043667\_contig\_number\_2925SRS053398\_contig\_number\_16038SRS075773\_contig\_number\_2786SRS1054716\_contig\_number\_9349SRS143598\_contig\_number\_373SRS147977\_contig\_number\_4597


SRS011134\_contig\_number\_13228SRS012273\_contig\_number\_24913SRS013098\_contig\_number\_11326SRS013215\_contig\_number\_647SRS014923\_contig\_number\_37345SRS016018\_contig\_number\_1564SRS018049\_contig\_number\_398SRS018133\_contig\_number\_3420SRS018984\_contig\_number\_4549SRS019286\_contig\_number\_1424SRS019685\_contig\_number\_8387SRS023346\_contig\_number\_2490SRS023715\_contig\_number\_1591SRS024549\_contig\_number\_470SRS024663\_contig\_number\_4955SRS042628\_contig\_number\_24255SRS043768\_contig\_number\_5422SRS048981\_contig\_number\_1593SRS052078\_contig\_number\_1327SRS054590\_contig\_number\_421SRS057717\_contig\_number\_1586SRS058770\_contig\_number\_2991SRS065504\_contig\_number\_23347SRS075021\_contig\_number\_89SRS075341\_contig\_number\_11960SRS077024\_contig\_number\_9656SRS098655\_contig\_number\_2553SRS101268\_contig\_number\_158SRS1041038\_contig\_number\_16501SRS1041112\_contig\_number\_2518SRS1055034\_contig\_number\_8479SRS1055043\_contig\_number\_1093SRS140645\_contig\_number\_1253SRS142712\_contig\_number\_4942SRS149784\_contig\_number\_17946SRS149879\_contig\_number\_24537SRS893230\_contig\_number\_7203
357276
species
37

246787
species
1

SRS048060\_contig\_number\_3017


SRS023526\_contig\_number\_64SRS971275\_contig\_number\_23803
2292944
2
species


SRS014613\_contig\_number\_852SRS064276\_contig\_number\_6388SRS1041145\_contig\_number\_22157SRS1055067\_contig\_number\_7082SRS1055099\_contig\_number\_2120
species
5
2292935


SRS077194\_contig\_number\_11293
1
species
2292938


SRS075078\_contig\_number\_11446
371601
species
1

1
family
2005519

1
genus
1348911


SRS143417\_contig\_number\_24768
species
1
1941478
